# The role of H1 antihistamines in contralateral breast cancer: a Danish nationwide cohort study

**DOI:** 10.1038/s41416-020-0747-4

**Published:** 2020-02-17

**Authors:** Annet Bens, Christian Dehlendorff, Søren Friis, Deirdre Cronin-Fenton, Maj-Britt Jensen, Bent Ejlertsen, Timothy L. Lash, Niels Kroman, Lene Mellemkjær

**Affiliations:** 1 Unit of Virus, Lifestyle and Genes, Danish Cancer Society Research Center, Copenhagen, Denmark; 2 Unit of Statistics and Pharmacoepidemiology, Danish Cancer Society Research Center, Copenhagen, Denmark; 3 Department of Public Health, University of Copenhagen, Copenhagen, Denmark; 4 Department of Clinical Epidemiology, Aarhus University Hospital, Aarhus, Denmark; 5 Danish Breast Cancer Group, Rigshospitalet, Copenhagen, Denmark; 6 Department of Oncology, Rigshospitalet, Copenhagen, Denmark; 7 Department of Epidemiology, Rollins School of Public Health, Emory University, Atlanta, GA USA; 8 Winship Cancer Institute, Emory University, Atlanta, GA USA; 9 Department of Breast Surgery, Herlev Hospital, Copenhagen, Denmark

**Keywords:** Breast cancer, Cancer epidemiology

## Abstract

**Background:**

Preclinical studies have shown both pro- and antineoplastic effects of antihistamines. Here, we evaluated the effect of H1 antihistamines on contralateral breast cancer (CBC) risk, and whether cationic amphiphilic (CAD) antihistamines could increase the sensitivity to chemotherapy.

**Methods:**

From the Danish Breast Cancer Group clinical database, we identified all women aged ≥20 years with a first-time diagnosis of breast cancer during 1996–2012. Information on drug use, CBC and potential confounding factors was retrieved from nationwide registries. Using Cox proportional hazard regression models, we calculated hazard ratios (HRs) and 95% confidence intervals (CIs) for CBC associated with H1-antihistamine use.

**Results:**

We identified 52,723 patients with breast cancer with a total of 310,583 person-years of follow-up. Among them, 1444 patients developed a new primary tumour in the contralateral breast. Post-diagnosis use of H1 antihistamines (≥2 prescriptions) was not strongly associated with CBC risk (HR 1.08, 95% CI: 0.90–1.31) compared with non-use (<2 prescriptions). Use of CAD antihistamines among patients receiving chemotherapy was not associated with a decrease in CBC risk.

**Conclusions:**

Taken together, our findings do not suggest any association of H1-antihistamine use with CBC development.

## Background

Accumulating evidence suggests that histamine may modulate proliferation of normal and malignant cells.^1^ Histamine plays a role in various physiological and pathological processes in the development and functioning of the mammary gland.^2,3^ Moreover, high levels of histamine have been detected in tumour tissue from breast cancer patients, and their levels of histamine in serum have been found to be higher than in healthy controls.^4^

Histamine 1 (H1)-receptor antihistamines, primarily used as first-line treatment for the relief of allergic symptoms, work as inverse agonists of the H1 receptor, which results in inhibition of mast cell activity and histamine release.^5^ The effect of H1 antihistamines on tumour development is equivocal. In the early 1990s, it was suggested that H1 antihistamines may increase the risk of breast cancer due to their structural similarity to N,N-diethyl-2-[4-(phenylmethyl) phenoxy] ethanamine (DPPE), a compound stimulating chemically induced mammary carcinoma in rodents.^6,7^ In contrast, during recent years, H1 antihistamines have gained interest due to reports of potential antineoplastic activity.^8–10^

To date, two epidemiological studies investigated the use of H1 antihistamines and breast cancer incidence.^11,12^ One study reported a slightly reduced risk,^11^ while the other found a null association.^12^ Nonetheless, the effects of H1 antihistamines on breast cancer risk may be more pronounced when studying contralateral breast cancer (CBC) risk among breast cancer survivors for whom susceptibility to breast cancer has already been proven. On the basis of studies on tamoxifen, it has been proposed that the occurrence of contralateral tumours among women with breast cancer may serve as a useful model for prevention.^13^ Furthermore, a subgroup of H1 antihistamines, the cationic amphiphilic drugs (CAD) antihistamines, has shown to increase the efficacy of chemotherapy.^14^ Adjuvant chemotherapy has previously been associated with reduced CBC risk,^15,16^ and this association may be more pronounced when it is administered simultaneously with CAD antihistamine use.

To increase the knowledge on whether H1 antihistamines affect breast cancer development, we examined the incidence of CBC among users of H1 antihistamines in a large cohort of Danish breast cancer survivors.

## Methods

This population-based cohort study was based on data from several Danish nationwide registries, including the Danish Breast Cancer Group (DBCG) clinical database,^17^ the National Prescription Registry,^18^ the National Patient Register,^19^ the Danish Cancer Registry,^20^ the Danish Pathology Register^21^ and registers on Statistics Denmark^22^ and the Civil Registration System.^23^ Detailed descriptions of the registries are available in the Supplementary Material of one of our previous publications.^24^ Unambiguous linkage between the registries was possible by using the personal identification number assigned to all Danish residents.

### Study population

The DBCG database is a nationwide, clinical database containing detailed information on nearly all women diagnosed with breast cancer in Denmark since 1977.^17^ We identified a cohort of women with a diagnosis of unilateral, non-metastatic breast cancer between 1996 and 2012. Breast cancer patients were eligible if they were 20 years or older at diagnosis, underwent surgery and had registered information on the laterality of the first breast cancer (*n* = 58,704). We excluded patients diagnosed with another malignancy (except non-melanoma skin cancer) at any time before the date of the first breast cancer diagnosis (*n* = 3344).

### Exposure definition

We identified data on the use of H1 antihistamines (ATC = R06A) from the National Prescription Registry, a register that captures data on all prescriptions dispensed at Danish pharmacies since 1995.^18^ Medications administered by hospitals (during admission or supplied by the hospital), treatment centres or certain institutions are not included in the registry.^18^ H1 antihistamines were further grouped according to CAD structure into CAD antihistamines, non-CAD antihistamines and mixed use, respectively. Users contributed person-years to the mixed user category when they switched from CAD to non-CAD antihistamines or vice versa. The specific ATC codes are available in Supplementary Table I.

We analysed H1-antihistamine use by applying two different models. In model 1, the time-varying model, use was defined as two or more prescriptions redeemed on separate dates after the first breast cancer diagnosis, and assessed as a time-varying covariate with a 1-year lag time. This means that after diagnosis, H1-antihistamine users were regarded as non-users until 1 year after their second prescription. In this time-varying model, we further investigated H1-antihistamine use according to cumulative amount and intensity of use. Cumulative amount was assessed as the total number of defined daily doses (DDDs) filled during follow-up and categorised into tertiles of <48, 48–300 and >300 DDDs. Intensity of use was defined as the cumulative amount of DDDs divided by the number of days between the first and latest prescription, and categorised into tertiles of <0.36, 0.36–0.95 and >0.95 DDDs/day. We also evaluated the influence of timing of H1-antihistamine use by an exposure matrix of four categories: (1) no pre- or post-diagnosis use (reference group), (2) pre-diagnosis use only, (3) pre- and post-diagnosis use (continuing users) and (4) post-diagnosis use only (new users). Pre-diagnosis use was defined as two or more prescriptions redeemed on separate dates within 2 years prior to the first breast cancer diagnosis.

In model 2, the baseline model, we evaluated H1-antihistamine use in a fixed exposure period to investigate the potential interaction with chemotherapy. Here, H1-antihistamine use was defined as ≥1 prescription and divided into two exposure windows: (1) 3 months before and 6 months following breast cancer diagnosis (early baseline use) and (2) 7–12 months following breast cancer diagnosis (late baseline use). Early baseline use was further divided according to CAD-structure and chemotherapy use. Even though antihistamines may be used daily in certain periods, they may also be used sporadically, and then prescriptions may last for several months. Therefore, we find it possible that tablets on a prescription up to 3 months before the breast cancer diagnosis may have been taken after the diagnosis during chemotherapy. In these analyses, we excluded all women with a first breast cancer during 2007–2012, since antihistamines are often used routinely at the hospital as premedication for taxane-based chemotherapy to prevent hypersensitivity reactions or to treat such reactions, and 90% of women receiving chemotherapy from 2007 were treated with taxanes.^25^

### Outcome and follow-up

Information on CBC was obtained from a previously established nationwide CBC database based on data from the Danish Cancer Registry^20^ and other registers. According to specific coding rules in the Cancer Registry, contralateral tumours with the same histology as the first breast cancer are not registered as new individual cancer records, but instead labelled as ‘bilateral breast cancer’. Therefore, we retrieved additional information on these CBCs through the original paper notification forms to the Cancer Registry for the period 1978–2003, and electronic records in the Danish Pathology Registry from 2004 and onwards,^21^ and only cases confirmed by these sources were accepted as CBCs in the database. Additional CBCs registered in the DBCG database only were added to obtain the highest degree of completeness (11% of all CBCs in the CBC Database).

From the Civil Registration System, we obtained information on death and emigration.^23^ The Danish Cancer Registry provided information on other cancer diagnoses.^20^ We used the National Patient Registry to identify women who received a mastectomy of the contralateral breast.^19^ In order to not overlook CBCs recorded after an accompanying mastectomy, we added 60 days to the date of the mastectomy, assuming that mastectomy without an accompanying CBC diagnosis within this time interval represented prophylactic mastectomy.

Follow-up in all analyses began 12 months after the first breast cancer diagnosis and ended at the occurrence of CBC, with censoring at the date of ipsilateral breast cancer, other primary malignancies (except non-melanoma skin cancer), mastectomy of the contralateral breast + 60 days (~prophylactic mastectomy), emigration and death or on December 31, 2013. Patients who developed CBC or any of the censoring outcomes within 12 months following the first breast cancer diagnosis were excluded (*n* = 2637).

### Information on potential confounders

We based the a priori selection of confounders on knowledge from the literature and availability in the nationwide registers. Information on age at diagnosis, year of diagnosis (1996–2000; 2001–2004; 2005–2008; 2009–2012), lobular histology (yes/no), oestrogen receptor (ER) status (positive; negative; unknown), lymph node status (positive; negative; unknown) and treatment modality of the first primary breast cancer was obtained from the DBCG Database.^17^ For treatment, we used an intention-to-treat variable, representing the prescribed treatment according to DBCG’s guidelines, and categorised it using a matrix combining endocrine, chemo- and radiation therapy (nine categories: endocrine therapy only; chemotherapy only; radiation therapy only; endocrine + chemotherapy; endocrine + radiation therapy; chemo- + radiation therapy; endocrine + chemo- + radiation therapy; no therapy; unknown). We used the National Patient Registry^19^ to retrieve information on diabetes, and alcohol-, tobacco- and allergy-related conditions during a period of 10 years before and until 1 year after the first breast cancer diagnosis (yes/no) (Supplementary Table I). Information on other concomitant drug use was obtained from the National Prescription Registry^18^ (Supplementary Table I). We included information on pre-diagnosis use of hormone-replacement therapy (≥2 prescriptions) and post-diagnosis use (≥2 prescriptions) of low-dose aspirin, high-dose aspirin, non-aspirin non-steroidal anti-inflammatory drugs (NSAID), statins, bisphosphonates, digoxin and metformin. Use of the post-diagnosis confounder drugs was modelled in the same way as H1 antihistamines in the time-varying model, and as first-year post-diagnosis use in the baseline model. Socio-economic status was measured by the highest achieved education (short: <9 years; medium: 10–12 years; high >12 years; unknown) at breast cancer diagnosis, and was obtained from the Danish Education Register at Statistics Denmark.^22^

### Statistical analysis

We calculated the proportions of breast cancer patients within categories of the covariates at baseline according to H1-antihistamine use (≥2 prescriptions) within the first 12 months following breast cancer diagnosis. By using Cox proportional hazard regression models, we estimated age- and multivariable-adjusted hazard ratios (HRs) and 95% confidence intervals (CIs) for H1-antihistamine use and CBC. The multivariable-adjusted model included the above-mentioned confounders in addition to age at the first breast cancer diagnosis (restricted cubic spline). Time since 1 year after the first breast cancer diagnosis was used as the underlying timescale.

In the time-varying model, we investigated effect measure modification by ER status and histological subtype of the first breast cancer. Moreover, we did the analysis according to ER status of the CBC, thereby only counting ER-positive CBCs as outcomes while censoring on all other CBCs and vice versa.

In post hoc sensitivity analyses of ever vs. non-use of H1 antihistamines and stratifications of use, we (1) removed the lag time and (2) extended the lag time to 2 years. The start of follow-up was unchanged, i.e. start date was 12 months after the first breast cancer diagnosis.

In the baseline model, we stratified according to CAD structure and chemotherapy (intention to treat: yes/no).

We evaluated the influence of competing events by calculating age- and multivariable-adjusted HRs and the corresponding 95% CIs for four censoring criteria (ipsilateral breast cancer, other primary malignancies, mastectomy of the contralateral breast and death) combined as one outcome variable.

In secondary analysis, we investigated potential bias by over-the-counter (OTC) use of H1 antihistamines in a multidimensional bias analysis as described by Lash et al.^26^ Assuming non-differential misclassification, a tabular quantitative bias analysis model was implemented by calculating bias-adjusted rate ratios using combinations of values assigned to the sensitivity, the probability of correctly classifying an exposed individual (0.7, 0.75, 0.8, 0.85, 0.9, 0.95 and 1.0) and specificity, the probability of correctly classifying an unexposed individual (0.95, 0.96, 0.97, 0.98, 0.99 and 1.0) (Supplementary Information and Supplementary Tables IIa, IIb and IIc). The specificity in our study was the probability among the true non-users of having no record of antihistamine use in the Prescription Register and was assumed to be high (≥98%).

The assumption of proportional hazards was evaluated by testing for trends in the Schoenfeld residuals in the fully adjusted model (ever use vs. non-use). All analyses were performed using R Statistical software version 3.4.2.^27^

## Results

This study included 52,723 patients diagnosed with breast cancer between 1996 and 2012 (Table 1). Overall, we identified 1444 patients diagnosed with a CBC over a total median follow-up of 4.8 years. Approximately 3.2% of the breast cancer patients (*n* = 1685) redeemed two or more H1-antihistamine prescriptions between breast cancer diagnosis and start of follow-up, and this proportion increased to 9.5% H1-antihistamine users (*n* = 5005) during follow-up. There were no major differences between H1-antihistamine users and non-users in the first year after breast cancer in terms of age, calendar period, main tumour characteristics and treatment of the first breast cancer (Table 1). H1-antihistamine users were more often diagnosed with comorbidities, such as smoking-related diseases and allergy-related conditions, and more likely to have used hormone-replacement therapy before diagnosis and non-aspirin NSAIDS in the first 12 months after diagnosis.

**Table 1:** Danish patients with breast cancer registered in the DBCG clinical database between 1996 and 2012, according to antihistamine use (≥2 prescriptions) within the first year following diagnosis.

	Non-users		First-year H1-antihistamine users	
	No.	%	No.	%
Total	51,038	100	1685	100
*Age at first BC, years*				
20–34	791	1.5	12	0.7
35–44	4606	9.0	114	6.8
45–54	11,569	23	362	22
55–64	15,286	30	519	31
65–74	11,488	23	454	27
≥75	7298	14	224	13
*Menopausal status at first BC*				
Premenopausal	12,970	25	377	22
Postmenopausal	37,380	73	1286	76
Unknown	688	1.3	22	1.3
*Calendar period of the first BC*				
1996–2000	13,298	26	353	21
2001–2004	11,239	22	356	21
2005–2008	11,919	23	424	25
2009–2012	14,582	29	552	33
*Histology of the first BC*				
Ductal	40,528	79	1348	80
Lobular	5502	11	178	11
Other	4670	9.2	142	8.4
Unknown	338	0.7	17	1.0
*Tumor size of the first BC*				
≤20 mm	30,047	59	1033	61
21–50 mm	18,001	35	559	33
≥51 mm	1670	3.3	51	3.0
Unknown	1320	2.6	42	2.5
*Lymph node status of the first BC*				
Negative	26,555	52	914	54
Positive	22,402	44	696	41
Unknown	2081	4.1	75	4.5
*ER status of the first BC*				
Positive	40,276	79	1317	78
Negative	9495	19	329	20
Unknown	1267	2.5	39	2.3
*Type of surgery at the first BC*				
Mastectomy	26,288	52	816	48
Breast-conserving surgery	24,750	48	869	52
*Adjuvant treatment for the first BC*				
* Endocrine therapy*				
Yes	26,544	52	884	53
No	18,611	37	629	37
Unknown	5883	12	172	10
* Chemotherapy*				
Yes	17,128	34	556	33
No	28,026	55	957	57
Unknown	5884	12	172	10
* Radiation therapy*				
Yes	31,535	62	1057	63
No	13,174	26	440	26
Unknown	6329	12	188	11
*Treatment matrix (combination of therapies)*				
No treatment	4334	8.5	133	7.9
Endocrine therapy only	5335	10	185	11
Chemotherapy only	2108	4.1	79	4.7
Radiation therapy only	6771	13	224	13
Endocrine- and chemotherapy	1397	2.7	43	2.6
Endocrine- and radiation therapy	11,304	22	407	24
Chemo- and radiation therapy	5271	10	189	11
Endocrine-, chemo- and radiation therapy	8187	16	236	14
Unknown	6331	12	189	11
*Comorbidities*				
Alcohol-related disease	807	1.6	42	2.5
Diabetes mellitus	1872	3.7	103	6.1
Tobacco-related disease	2498	4.9	259	15
Allergy-related conditions	1304	2.6	152	9.0
*Other drug exposures*				
* Pre-diagnosis*				
Hormone-replacement therapy	12,128	24	638	38
* First-year post diagnosis*				
Low-dose aspirin	5069	9.9	226	13
High-dose aspirin	70	0.1	17	1.0
Non-aspirin NSAIDs	5782	11	360	21
Statins	5243	10	238	14
Bisphosphonates	1697	3.3	75	4.5
Metformin	1212	2.4	60	3.6
Digoxin	1069	2.1	34	2.0
*Highest achieved education at the first BC*				
Short	12,635	25	427	25
Medium	23,179	45	749	45
High	11,977	23	413	25
Unknown	3247	6.4	96	5.7

*BC* breast cancer, *DBCG* Danish Breast Cancer Group, *ER* oestrogen receptor, *NSAID* non-steroidal anti-inflammatory drug.

Ever use of H1 antihistamines was not strongly associated with risk of CBC (HR: 1.08, 95% CI: 0.90–1.31) (Table 2). The HRs did not change substantially by cumulative dose or CAD structure. Low- and medium-intensity H1-antihistamine use were associated with HRs close to unity, while high-intensity use was associated with a HR of CBC of 1.45 (95% CI: 1.00–2.12). Associations were similar among patients who had used H1 antihistamines exclusively after (new users) or both before and after (continuing users) the first breast cancer diagnosis, compared with non-users (Table 2). In post hoc sensitivity analyses, we found no major changes in these risk estimates when removing the lag time or extending it to 2 years (Supplementary Table III).

**Table 2:** Association between time-varying post-diagnosis H1-antihistamine use (≥2 prescriptions) and risk of CBC among Danish patients with breast cancer.

	No.	Person-years	No. of CBC cases	Age-adjusted HR (95% CI)	Multivariable-adjusted^a^ HR (95% CI)
Non-use	52,723	286,258	1317	1	1
Ever use	5005	24,325	127	1.07 (0.89–1.29)	1.08 (0.90–1.31)
*Cumulative amount (DDDs)*					
Non-use	52,723	286,258	1317	1	1
Low (<48.32 DDDs)	3704	9011	46	1.04 (0.77–1.39)	1.04 (0.77–1.39)
Medium (48.32–300 DDDs)	3351	8675	42	1.00 (0.73–1.36)	1.01 (0.74–1.37)
High (>300 DDDs)	1625	6639	39	1.20 (0.87–1.65)	1.26 (0.91–1.75)
*Intensity (DDDs/day)*					
Non-use	52,723	286,258	1317	1	1
Low (<0.36 DDDs/day)	3186	14,104	73	1.04 (0.82–1.32)	1.04 (0.82–1.32)
Medium (0.36–0.95 DDDs/day)	1924	5853	26	0.93 (0.63–1.37)	0.94 (0.64–1.39)
High (>0.95 DDDs/day)	1640	4368	28	1.36 (0.93–1.97)	1.45 (1.00–2.12)
*CAD structure*					
Non-use	52,723	286,258	1317	1	1
CAD antihistamine	1471	6059	32	1.08 (0.76–1.54)	1.07 (0.75–1.52)
Non-CAD antihistamine	3526	14,722	77	1.07 (0.85–1.35)	1.10 (0.87–1.39)
Mixed^b^	788	3544	18	1.02 (0.64–1.62)	1.03 (0.65–1.65)
*Timing*^c^					
Non-use	47,530	256,164	1156	1	1
Pre-diagnosis use only	2557	6213	22	0.81 (0.53–1.24)	0.82 (0.54–1.25)
Post-diagnosis use only (new use)	3043	13,513	70	1.05 (0.82–1.35)	1.05 (0.82–1.35)
Pre- and post-diagnosis use (continued use)	1620	8168	40	1.05 (0.76–1.44)	1.08 (0.78–1.48)

*CAD* cationic amphiphilic drugs, *CBC* contralateral breast cancer, *CI* confidence interval, *HR* hazard ratio.

^a^Adjusted for age at the first breast cancer, calendar period at the first breast cancer, lobular histology of the first breast cancer, ER status of the first breast cancer, lymph node status of the first breast cancer, therapy for the first breast cancer (endocrine therapy only, chemotherapy only, radiation therapy only, endocrine + chemotherapy, endocrine + radiation therapy, chemo- + radiation therapy, endocrine + chemo- + radiation therapy, no therapy and unknown therapy), pre-diagnosis use of hormone-replacement therapy, time-dependent post-diagnosis use of low- and high-dose aspirin, non-aspirin NSAIDs, bisphosphonates, metformin and digoxin, alcohol-related disease, diabetes mellitus, tobacco-related disease, allergy-related conditions and highest achieved education at the first breast cancer diagnosis.

^b^Mixed use was defined as switching from CAD to non-CAD antihistamine use or vice versa.

^c^Restricted to patients with a first breast cancer diagnosed during 1997 and 2012. In this analysis, the non-use category includes exclusively patients with < 2 H1-antihistamine prescriptions and no records of pre-diagnosis use.

Stratification by ER status and histology did not substantially influence the associations (Table 3). The HRs for ER-positive and ER-negative CBC were 1.01 (95% CI: 0.81–1.27) and 1.18 (95% CI: 0.74–1.86), respectively.

**Table 3:** Association between time-varying post-diagnosis H1-antihistamine use (≥2 prescriptions) and risk of CBC among Danish patients with breast cancer, stratified by ER status and histology of the first breast cancer and ER status of CBC.

	No.	Person-years	No. of CBC cases	Age-adjusted HR (95% CI)	Multivariable-adjusted^a^ HR (95% CI)
*ER status of the first BC*					
* ER-positive*					
Non-use	41,593	224,388	972	1	1
Ever use	3946	18,658	97	1.10 (0.89–1.36)	1.12 (0.90–1.39)
* ER-negative*					
Non-use	9824	52,301	301	1	1
Ever use	888	4648	22	0.85 (0.55–1.31)	0.86 (0.56–1.34)
*Histology of the first BC*					
* Ductal*					
Non-use	41,876	227,750	1010	1	1
Ever use	4018	19,728	97	1.05 (0.85–1.29)	1.06 (0.85–1.31)
* Lobular*					
Non-use	5680	32,235	177	1	1
Ever use	533	2471	18	1.29 (0.79–2.11)	1.31 (0.80–2.15)
*ER status of CBC*					
* ER-positive*					
Non-use	52,723	286,258	896	1	1
Ever use	5005	24,325	87	0.99 (0.79–1.24)	1.01 (0.81–1.27)
* ER-negative*					
Non-use	52,723	286,258	230	1	1
Ever use	5005	24,325	21	1.18 (0.75–1.86)	1.18 (0.74–1.86)

*BC* breast cancer, *CBC* contralateral breast cancer, *CI* confidence interval, *HR* hazard ratio.

^a^Adjusted for age at the first breast cancer, calendar period at the first breast cancer, lobular histology of the first breast cancer, ER status of the first breast cancer, lymph node status of the first breast cancer, therapy for the first breast cancer (endocrine therapy only, chemotherapy only, radiation therapy only, endocrine + chemotherapy, endocrine + radiation therapy, chemo- + radiation therapy, endocrine + chemo- + radiation therapy, no therapy and unknown therapy), pre-diagnosis use of hormone-replacement therapy, time-dependent post-diagnosis use of low- and high-dose aspirin, non-aspirin NSAIDs, bisphosphonates, metformin and digoxin, alcohol-related disease, diabetes mellitus, tobacco-related disease, allergy-related conditions and highest achieved education at the first breast cancer diagnosis.

The association between H1-antihistamine use and CBC at baseline is given in Table 4, divided into an early baseline period of 3 months before until 6 months after diagnosis, and a late baseline period of 7–12 months after diagnosis. We observed HRs for CBC of 1.02 (95% CI: 0.80–1.29) and 1.42 (95% CI: 1.00–1.98) for a post-diagnosis H1-antihistamine prescription being filled in the period of 3 months before and 6 months following and 7–12 months following breast cancer diagnosis, respectively. In contrast to our hypothesis, although based on small numbers, when stratifying according to chemotherapy at the first breast cancer diagnosis, the HR for CBC was slightly higher among CAD antihistamine users who received chemotherapy compared with those who did not.

**Table 4:** Association between H1-antihistamine use (≥1 prescription) within 3 months prior to and 1 year following diagnosis and risk of CBC among Danish patients with breast cancer diagnosed between 1996 and 2006.

	No.	Person-years	No. of CBC cases	Age-adjusted HR (95% CI)	Multivariable-adjusted^a^ HR (95% CI)
Non-use	28,532	231,011	1,138	1	1
Early baseline use (–3 to 6 months)	1808	14,813	75	1.02 (0.81–1.29)	1.02 (0.80–1.29)
Late baseline use (7–12 months)^b^	610	4838	34	1.42 (1.01–2.00)	1.42 (1.00–1.98)
*Chemotherapy*^c^					
Non-use	7159	58,349	289	1	1
* Early baseline use (–3 to 6 months)*					
CAD antihistamines	184	1549	11	1.39 (0.76–2.55)	1.30 (0.71–2.39)
Non-CAD antihistamines	298	2567	12	0.94 (0.53–1.68)	0.88 (0.49–1.58)
Mixed^d^	10	71	0	–	–
*No chemotherapy*^c^					
Non-use	15,885	137,806	710	1	1
* Early baseline use (–3 to 6 months)*					
CAD antihistamines	366	3226	–^e^	0.77 (0.45–1.34)	0.81 (0.47–1.40)
Non-CAD antihistamines	623	5266	–^e^	1.00 (0.68–1.46)	1.03 (0.70–1.52)
Mixed^f^	39	342	–^e^	–	–

*CAD* cationic amphiphilic drugs, *CBC* contralateral breast cancer, *CI* confidence interval, *HR* hazard ratio.

^a^Adjusted for age at the first breast cancer, calendar period at the first breast cancer, lobular histology of the first breast cancer, ER status of the first breast cancer, lymph node status of the first breast cancer, therapy for the first breast cancer (endocrine therapy only, chemotherapy only, radiation therapy only, endocrine + chemotherapy, endocrine + radiation therapy, chemo- + radiation therapy, endocrine + chemo- + radiation therapy, no therapy and unknown therapy), pre-diagnosis use of hormone-replacement therapy, first-year post-diagnosis use of low- and high-dose aspirin, non-aspirin NSAIDs, bisphosphonates, metformin and digoxin, alcohol-related disease, diabetes mellitus, tobacco-related disease, allergy-related conditions and highest achieved education at the first breast cancer diagnosis.

^b^Exclusively women who started using H1 antihistamines during 7–12 months following the first breast cancer diagnosis.

^c^Intention to treat: chemotherapy prescribed according to the Danish Breast Cancer Group guidelines (chemotherapy status unknown for 5937 breast cancer patients).

^d^Mixed use was defined as switching from CAD to non-CAD antihistamine use or vice versa.

^e^Small numbers not presented for protection of privacy in accordance with the data security policy of Statistics Denmark.

In the analyses on competing events, HRs for the combined outcome were similar to those for CBC (results not shown).

The quantitative bias analysis showed that OTC use of H1 antihistamines resulted in a bias towards the null (Supplementary Table IIc). However, as the exposure prevalence of H1-antihistamine use is rare (~10%), the magnitude of the bias is mainly influenced by the specificity.

## Discussion

In this population-based cohort study of breast cancer patients, we found no support of an association between use of H1 antihistamines and CBC development, neither overall nor within specific classes of antihistamines. This finding was consistent after stratification by ER status and histology of the first breast cancer. Further, we did not observe a reduction in CBC risk for CAD antihistamine use when prescribed closely in time to the course of chemotherapy.

Conflicting results have emerged on breast cancer growth from preclinical studies regarding H1 antihistamines. Originally, H1 antihistamines have long been seen as potential risk factors for cancer as their amino-ethyl group has structural similarity to DPPE.^6,7^ More recently, preclinical research has pointed towards potential anticancer properties. Two experimental studies by Garcia-Quiroz et al. demonstrated the capacity of astemizole, an old second-generation H1 antihistamine, to inhibit breast cancer tumour growth in vitro^28^ and in vivo^29^ by targeting Eag1, a potassium ion channel involved in tumour progression. Just recently, Fernandez-Nogueira et al. found the H1 receptor to be overexpressed in basal and HER2-targeted therapy-resistant breast cancer cells, and demonstrated that inhibition of the H1 by terfenadine led to increased cell death and a reduction in cell proliferation, although only in these two specific types of breast cancer.^9^ H1 antihistamines have also been shown to exert growth-inhibitory effects and promote apoptosis in vitro in other cancer cell lines.^10,30–32^

One previous study investigating breast cancer risk does to some extent support the anticancer effects found in preclinical studies. This case–control study from the United States by Kelly et al.,^11^ included almost 6000 cases with invasive breast cancer, and reported an OR of 0.8 (95% CI: 0.6–1.0) for regular antihistamine use and risk of breast cancer. A Canadian study by Nadalin et al. based on 3133 breast cancer cases observed no effect of antihistamine use on breast cancer risk (OR: 0.93, 95% CI: 0.81–1.06).^12^ The overall null associations observed in our study are in line with findings in the latter study. Both previous studies found no evidence of a duration–response pattern.^11,12^ Our results pointed towards a cancer-promoting effect with increasing intensity of use, though we saw no evidence of a dose–response association, so this finding may be due to chance.

The hypothesis that CAD antihistamines may improve the response to chemotherapy has been proposed already in 1993 by Hait et al., who reported on the ability of terfenadine to increase sensitisation of a multidrug-resistant human breast cancer cell line to the chemotherapeutic drug, doxorubicin.^33^ This finding is compatible with a recently published study from our institution, where HRs for all-cause mortality associated with use of the CAD antihistamines, loratidine and desloratidine, were found to be lower among patients diagnosed with advanced cancer who received chemotherapy compared with those who did not.^14^ Furthermore, they reported on the ability of low concentrations of CAD antihistamines to increase the sensitivity to chemotherapy in triple-negative breast cancer cells in vitro.^14^ We only had few cases of CBC who had used CAD- or non-CAD antihistamines during the period of chemotherapy administration, and therefore can neither support nor reject the hypothesis that the response to chemotherapy will be strengthened by use of CAD antihistamines.

Besides being the first to investigate the effect of H1 antihistamines in a population at high risk of breast cancer, another major strength of our study includes its nationwide approach covering the entire population of women with breast cancer in Denmark. Second, we solely used continuously updated, high-quality registries, which gave us virtually complete follow-up and accurate, prospectively collected data on H1-antihistamine use and potential confounding factors. Third, we ensured high case validity of the CBCs by combining data from the Danish Cancer Registry, the Pathology Register, the original paper notification forms and the DBCG database.

Our study, however, has some limitations. We did not have information available on OTC use of H1 antihistamines, which may have introduced exposure misclassification bias. During the study period, around 75% of the purchased H1 antihistamines were redeemed on prescription; for the main H1 antihistamines, this was 55% for cetirizine, 65% for loratadine, 97% for fexofenadine and >99% for desloratadine.^34^ As breast cancer patients typically are under close medical surveillance, these proportions may be even higher. Furthermore, the quantitative bias analysis suggested that the impact of the bias due to OTC use is likely to be negligible. Use of medicine at hospitals is also not recorded by the Prescription Register. To take into account that H1 antihistamines often used at the hospital to prevent or treat hypersensitivity reactions from taxane-based chemotherapy are not captured in the register, we excluded all women with the first breast cancer during 2007–2012 in our baseline model, when 90% of chemotherapy was taxane-based.^25^ Although our cohort included over 52,000 breast cancer patients, the number of CBC cases using H1 antihistamines was relatively limited. As a result, we were unable to examine use of the individual H1 antihistamines separately, and the risk estimates should be interpreted with caution. Lastly, residual confounding may arise from inclusion of missing values for some of the covariates in our multivariable model. Also, despite controlling for a broad range of covariates, we lacked information on some risk factors for CBC such as family history and pathogenic *BRCA1/2* mutations, but we find it unlikely that these factors are associated with the use of H1 antihistamines.

In summary, the use of H1 antihistamines after breast cancer diagnosis did not seem to affect the risk of a new contralateral tumour.

## Supplementary information

Supplementary Material

## Data Availability

The study was approved by the Danish Data Protection Board. Data were available from the Danish Breast Cancer Group (http://www.dbcg.dk/), Statistics Denmark (https://www.dst.dk/en/TilSalg/Forskningsservice) and the Research Service of Sundhedsdatastyrelsen (https://sundhedsdatastyrelsen.dk/da/forskerservice). R-code of analyses is available from the corresponding author upon request.
