# Supplementary material for: The role of H1 antihistamines in contralateral breast cancer: a Danish nationwide cohort study
Source: Br J Cancer. 2020 Feb 17;122(7):1102–8. doi: 10.1038/s41416-020-0747-4 (PMC7109031; doi:10.1038/s41416-020-0747-4)
Supplement: Supplementary file 1 — Supplementary Material [file 41416_2020_747_MOESM1_ESM.pdf]

Supplementary Material

The role of H1-antihistamines in contralateral breast cancer: a Danish nationwide cohort study

Annet Bens, Christian Dehlendorff, Søren Friis, Deirdre Cronin-Fenton, Maj-Britt Jensen,

Bent Ejlertsen, Timothy L Lash, Niels Kroman and Lene Mellemkjær

**Supplementary Table I. Registers and corresponding codes**	Page 2
**Supplementary Information and Tables IIa, IIb and IIc. Simple quantitative bias analysis**	Page 3
**Supplementary Table III. Extra analysis**	Page 4
**References**	Page 5

Supplementary Table I. Registers and corresponding codes

**The National Prescription Registry(1)** *Anatomical Therapeutic Classification codes*		
**Antihistamines**	**ATC**	
CAD antihistamines	R06AA04, R06AX11, R06AX12, R06AX13, R06AX22, R06AX27	
Non-CAD antihistamines	R06AA02, R06AB02, R06AD01, R06AD02, R06AD03, R06AD04, R06AE04, R06AE05, R06AE07, R06AE09, R06AX02, R06AX18, R06AX25, R06AX26, R06AX28, R06AX29	
**Low-dose aspirin**	B01AC06 (75, 100 and 150mg tablets)	
**High-dose aspirin**	N02BA51, N02BA01 (500mg tablets)	
**Non-aspirin NSAIDs**	M01A except M01AX	
**Statins**	C10AA, C10BA02	
**Bisphosphonates**	M05BA, M05BB	
**Metformin**	A10BA02	
**Digoxin**	C01AA05	
**Hormone replacement therapy**	G03C, G03D, G03F, G03HB01	
**The National Patient Registry(2)** *International Classification for Diseases (ICD) codes*		
	**ICD-8**	**ICD-10**
**Alcohol-related disease**	291, 303, 571.10, 577.10, 979, 980	F10, G31.2, G62.1, G72.1, I42.6, K29.2, K70, K86.0, R78.0, T51, Z72.1
**Smoking-related disease**	491, 492, 493	J41-J46
**Diabetes**	249, 250	E10, E11
**Allergy-related conditions**	507, 360.03, 692	J30, H10.1, L23-L25
**The National Patient Registry(2)** *Procedure codes*		
**Mastectomy**	KHAC	

Simple quantitative bias analysis

We performed a simple quantitative bias analysis to address the potential bias by over-the-counter (OTC) use of H1-antihistamines (exposure misclassification) according to the methods described by Lash et al. (2009)(3). Briefly, we calculated bias adjusted rate ratios (RRs) assigning varying values to the following bias parameters: 1. sensitivity, the probability of correctly classifying an exposed individual, and 2. specificity, the probability of correctly classifying an unexposed individual, under the assumption of non-differential misclassification. OTC use of H1-antihistamines mainly influences the sensitivity but not the specificity. The specificity in our study is influenced by the probability among the true non-users of having no record of antihistamine use in the Prescription Register and is assumed to be high (≥98%).

Supplementary Table IIa. Observed data

	H1-antihistamine user	Non-user	Total
**No. of CBC**	a = 127	b = 1313	$a + b$
**Person years**	c = 24,325	d = 286,258	$c + d$

Supplementary table IIb. Equations for calculating the expected data from the observed data using specificity (SP) and sensitivity (SE*)*

	H1-antihistamine user	Non-user
**No. of CBC**	$A = \frac{(\mathrm{a} - (1 - \mathrm{SP}) * (\mathrm{a} + \mathrm{b}))}{(\mathrm{SE} - (1 - \mathrm{SP}))}$	$B = (a + b) - A$
**Person-years**	$C = \frac{(\mathrm{c} - (1 - \mathrm{SP}) * (\mathrm{c} + \mathrm{d}))}{(\mathrm{SE} - (1 - \mathrm{SP}))}$	$D = (c + d) - C$

Supplementary table IIc. Bias adjusted RRs with varying levels of specificity and sensitivity

		Specificity					
		**0.95**	**0.96**	**0.97**	**0.98**	**0.99**	**1**
**Sensitivity**	**0.7**	1,356	1,265	1,212	1,176	1,151	1,133
	**0.75**	1,355	1,263	1,210	1,175	1,150	1,132
	**0.8**	1,355	1,262	1,209	1,174	1,148	1,131
	**0.85**	1,352	1,261	1,208	1,172	1,147	1,130
	**0.9**	1,351	1,260	1,207	1,172	1,146	1,129
	**0.95**	1,350	1,259	1,206	1,171	1,146	1,128
	**1.0**	1,349	1,258	1,205	1,170	1,145	1,127[a]

All bias adjusted RRs are multiplied by the relative risk due to confounding (HR_{crude} / $HR_{adjusted}$ = 1.07 / 1.08 = 0.99).

[a] Crude RR as calculated from the observed data

Supplementary Table III. Association between time-varying post-diagnosis H1-antihistamine use (≥2 prescriptions) and risk of CBC among Danish patients with breast cancer including no lag time or 2 years lag time

	0 year lag time				2 year lag time			
	**No.**	**Person-years**	**No. of CBC cases**	**Multivariable-adjusted HR (95% CI)[a]**	**No.**	**Person-years**	**No. of CBC cases**	**Multivariable-adjusted HR (95% CI)[a]**
Non use	51,038	281,750	1308	1	52,723	290,895	1340	1
Ever use	5864	28,833	136	0.98 (0.82–1.17)	4260	19,688	104	1.08 (0.88-1.33)
**Cumulative amount (DDDs)**								
Non use	51,038	281,750	1308	1	52,723	290,895	1340	1
Low	3678	10,493	49	0.95 (0.71-1.26)	3174	7393	41	1.11 (0.81-1.53)
Medium	3734	10,021	41	0.85 (0.62-1.16)	2814	6774	29	0.88 (0.60-1.27)
High	1935	8320	46	1.19 (0.88-1.60)	1406	5522	34	1.31 (0.93-1.86)
**Intensity (DDDs/day)**								
Non use	51,038	281,750	1308	1	52,723	290,895	1340	1
Low	3618	16,781	76	0.90 (0.72-1.14)	2690	11,332	60	1.05 (0.86-1.36)
Medium	2168	6967	32	0.98 (0.68-1.39)	1638	4815	20	0.87 (0.56-1.36)
High	1901	5085	28	1.25 (0.86-1.83)	1382	3540	24	1.53 (1.02-2.29)
**CAD-structure**								
Non use	51,038	281,750	1308	1	52,723	290,895	1340	1
CAD antihistamine	1613	6937	33	0.97 (0.68-1.37)	1282	5018	30	1.19 (0.83-1.72)
Non-CAD antihistamine	4172	17,509	80	0.96 (0.77-1.21)	2972	11,840	63	1.11 (0.86-1.43)
Mixed[b]	951	4387	23	1.06 (0.70-1.60)	640	2829	11	0.78 (0.43-1.43)
**Timing[c]**								
Non use	47,039	253,039	1148	1	47,530	258,956	1167	1
Pre-diagnosis use only	1434	5155	21	0.92 (0.60-1.41)	2557	7727	29	0.87 (0.60-1.26)
Post-diagnosis use only (new use)	3729	16,638	78	0.96 (0.76-1.21)	2548	10 721	59	1.11 (0.85-1.45)
Pre- and postdiagnosis use (continued use)	1749	9226	41	0.98 (0.72-1.35)	1394	6654	33	1.08 (0.76-1.53)

[a] Adjusted for age at first breast cancer, calendar-period at first breast cancer, lobular histology of first breast cancer, ER status of first breast cancer, lymph node status of first breast cancer, therapy for first breast cancer (endocrine therapy only, chemotherapy only, radiation therapy only, endocrine + chemotherapy, endocrine + radiation therapy, chemo- + radiation therapy, endocrine + chemo- + radiation therapy, no therapy and unknown therapy), pre-diagnosis use of hormone-replacement therapy, first-year post-diagnosis use of low and high-dose aspirin, non-aspirin NSAIDs, bisphosphonates, metformin and digoxin, alcohol-related disease, diabetes mellitus, tobacco-related disease, allergy-related conditions and highest achieved education at first breast cancer diagnosis.

References

1. Pottegard A, Schmidt SAJ, Wallach-Kildemoes H, Sorensen HT, Hallas J, Schmidt M. Data Resource Profile: The Danish National Prescription Registry. International journal of epidemiology. 2017;46(3):798-f.

2. Schmidt M, Schmidt SA, Sandegaard JL, Ehrenstein V, Pedersen L, Sorensen HT. The Danish National Patient Registry: a review of content, data quality, and research potential. Clinical epidemiology. 2015;7:449-90.

3. Lash TLF, M.P. Fink, A.K. Applying Quantitative Bias Analysis to Epidemiological Data. New York Springer 2009
